# Maternal Genistein Intake Mitigates the Deleterious Effects of High-Fat Diet on Glucose and Lipid Metabolism and Modulates Gut Microbiota in Adult Life of Male Mice

**DOI:** 10.3389/fphys.2019.00985

**Published:** 2019-07-30

**Authors:** Liyuan Zhou, Xinhua Xiao, Qian Zhang, Jia Zheng, Mingqun Deng

**Affiliations:** Department of Endocrinology, Key Laboratory of Endocrinology, Translational Medicine Center, Ministry of Health, Peking Union Medical College, Peking Union Medical College Hospital, Chinese Academy of Medical Sciences, Beijing, China

**Keywords:** maternal genistein intake, glucose metabolism, lipid metabolism, gut microbiota, adult life, male offspring, high-fat diet

## Abstract

Adverse early-life exposures program increased risk of chronic metabolic diseases in adulthood. However, the effects of genistein supplementation in early life on metabolic health in later life are largely unclear. Our objective was to investigate whether maternal genistein intake could mitigate the deleterious influence of a maternal high-fat diet on glucose and lipid metabolism in offspring and to explore the role of gut microbiota in mediating the transgenerational effects. C57BL/6 female mice were fed either a high-fat diet (HF), high-fat diet with genistein (0.6 *g*/kg diet) (HFG) or normal control diet (C) for 3 weeks before pregnancy and throughout pregnancy and lactation. The male offspring had *ad libitum* access to normal chow diet from weaning to 24 weeks of age. Then the content of inguinal subcutaneous adipose tissue (SAT) and epididymal visceral adipose tissue (VAT) were weighed. Glucose tolerance test (GTT), the level of serum insulin and lipid profiles were analyzed. The caecal contents were collected for 16S rDNA sequencing. The results showed that maternal genistein intake could significantly reduce blood glucose levels during GTT, fasting insulin levels, VAT mass and serum triglyceride levels as well as increase high-density lipoprotein cholesterol in adult male offspring. Significant decrease of germs from the Tenericutes phylum and enrichment of *Rikenella* as well as SCFA (short-chain fatty acid)-producing bacteria, including *Alloprevotella, Odoribacter*, and *Clostridium XlVa*, in offspring of genistein fed dams might play crucial roles in the improvement of glucose and lipid metabolism. Overall, early-life genistein intake attenuated the harmful effects of maternal HF on metabolism in adult offspring and the protective effects were associated with the alterations in gut microbiota, which provides new evidence and targets for mitigate the poor effects of adverse early-life exposures on metabolic health in later life.

## Introduction

Type 2 diabetes mellitus (T2DM) and obesity arise at the interface of genetics and environmental factors, including physical inactivity, smoking and nutrition intake. Interestingly, a large number of human studies and experimental animal models demonstrated that adverse environmental exposures experienced by their parents during intrauterine or early postnatal life significantly influenced health of the next generations (Dong et al., 2019; Lund et al., 2019; Zhang et al., 2019). More specifically, poor early-life exposures robustly increased the risks of developing chronic metabolic diseases in adult life, including glucose intolerance, T2DM and obesity (Csongova et al., 2018; Kearney et al., 2018; Wen et al., 2018; Yu et al., 2018; Chang et al., 2019). This association has been conceptualized by the fetal programming hypothesis and the developmental origins of health and disease, which propose that environmental stimuli during critical windows of development programmed permanent changes in later life (Aris et al., 2018; Velazquez et al., 2019). Although epigenetics, microbiome, and metabolome were all considered the potential mechanisms, the specific mechanism of the “fetal programming” is still largely unclear (Wankhade et al., 2016). Substantial research and our previous studies both showed that early-life overnutrition-maternal high-fat diet during pregnancy and lactation significantly increased susceptibility to glucose intolerance, insulin resistance and lipid disorders in adult offspring (Zheng et al., 2016; Huang et al., 2017; Thompson et al., 2019). Therefore, early-life might be a critical window for preventing the transmission of metabolic diseases across generation and reducing the prevalence of T2DM. Effective measures should be taken to reset the trajectories of chronic metabolic disease.

In recent years, bioactive food components have gained increasing attention in academia. Genistein, which is one of the most important ingredients in soy isoflavone, is structurally similar with 17β-estradiol and is also known as a phytoestrogen. The beneficial effects of genistein on cardiovascular disease (Shi et al., 2019), cancer (Lepri et al., 2018), bone metabolic disease (Ye et al., 2018) and Alzheimer’s disease (Devi et al., 2017) have been extensively researched. And the safety of genistein intake was also verified in both animals and humans (Gilbert and Liu, 2013). In terms of metabolic diseases, several large epidemiological studies in American, Japan, and Chinese have shown that the intake of soy isoflavone is negatively correlated with the risk of T2DM, especially in obese population (Nanri et al., 2010; Odegaard et al., 2011; Ding et al., 2016). Subsequently, growing numbers of clinical research and animal models confirmed the benefits of genistein on glucose intolerance, insulin sensitivity and lipid metabolic disorders (Gilbert and Liu, 2013; Liu et al., 2017). Evidence has indicated that the potential mechanisms deciphering the protective effects of genistein against metabolic disturbances included directly acting on β-cell to enhance proliferation, promoting secretion of insulin and inhibiting apoptosis, regulating glucose and lipid metabolism in liver, epigenetic modifications, modulating cAMP/PKA signaling pathway as well as modifying gut microbiota (Gilbert and Liu, 2013). However, studies exploring the effects of genistein intake on transgenerational metabolic health are lacking.

There is a community of 1000 or more species of bacteria that is 10 times more than human cells colonizing in gastrointestinal tract, which has multiple functions for the body. During the last few decades, emerging research has focused on the role of gut microbiota in metabolic diseases and showed that gut microbiota dysbiosis was intertwined with various disorders, such as glucose intolerance, insulin sensitivity and disturbances of serum lipid profiles (Mithieux, 2018). Recently, with the propose of “in utero colonization hypothesis,” increasing studies have demonstrated that gut microbiota might play a key role in the effects of adverse early-life exposures on metabolism in later life (Soderborg et al., 2016; Zhou and Xiao, 2018). And recent evidence has indicated the association between genistein intake and changes of gut microbiota (Lopez et al., 2018). However, the effects of genistein intake experienced by mothers on gut microbiota and the relationship between metabolic alterations and gut microbial communities in adult offspring are unclear.

Therefore, we aimed to explore whether maternal genistein intake for 3 weeks before pregnancy, and throughout pregnancy and lactation could mitigate the deleterious effects of the high-fat diet on glucose and lipid metabolism in adult offspring. In addition, to investigate the associated mechanisms, the changes of gut microbiota and the relationship between metabolic parameters and altered bacteria were also detected in our study.

## Materials and Methods

### Animals and Study Design

We obtained four-week-old C57BL/6 female mice from the National Institutes for Food and Drug Control (Beijing, China; SCXK-2014-0013). Mice were kept under SPF (specific pathogen free) conditions (room temperature at 22 ± 2°C; 12 h light/dark cycle). Mice had *ad libitum* access to food and sterile water throughout the study. After 1 week of environmental adaptation, dams were randomly assigned to three groups and were fed a high-fat diet (HF, *n* = 8), high-fat diet with genistein (CAS: 466-72-0, G0272, TCI Development Co., Ltd.) (0.6 *g*/kg diet) (HFG, *n* = 7) or normal control diet (AIN-93G) (C, *n* = 8) for 3 weeks. Previous studies have confirmed the metabolic protects of genistein at this dose (Al-Nakkash et al., 2012; Simperova et al., 2016). The ingredients are shown in Supplementary Table S1. The high-fat diet included 60% (kcal%) fat, whereas the control diet contained 15.8% fat.

After 3 weeks of intervention, females were mated to eight-week-old normal C57BL/6 males. The dams were checked for postcopulatory plugs every morning after mating, and the appearance of a plug was taken as d 0.5 of pregnancy. The pregnant females continued on their respective diets throughout the pregnancy and lactation. The litter size was all culled to 5 pups to ensure that there was no nutritional bias between litters. Offspring were weaned at 3 weeks of age. At weaning, all male offspring (*n* = 7–8 per group) were given *ad libitum* access to a normal chow diet until 24 weeks of age. The body weights of both the mothers and offspring were measured once per week. At the end of the experiment, one male offspring from different litters (*n* = 7–8 per group) were euthanized. Blood samples were collected from the intraorbital retrobulbar plexus after 10 h of fasting from anesthetized mice, and the inguinal subcutaneous adipose tissue **(SAT)** and epididymal visceral adipose tissue **(VAT)** were removed and weighed; the caecal contents were quickly removed, snap frozen in dry ice, and then stored at -80°C for further analysis. All the procedures were approved by the Animal Care and Use Committee of the Peking Union Medical College Hospital (Beijing, China, SYXC-2014-0029). All the animal operations were conducted in compliance with the National Institutes of Health Guide for the Care and Use of Laboratory Animals.

### Oral Glucose Tolerance Tests (OGTT)

The male offspring were fasted for 6 h and given a glucose load (2 g/kg of body weight) by gavage. Blood glucose (BG) levels were measured at 0, 30, 60, and 120 min after the gavage from tail vein blood using a Contour TS glucometer (ACCU-CHEK Mobile, Beijing, China). The area under the curve (AUC) of the OGTT was calculated as previously described (Zheng et al., 2014).

### Serum Biochemical Parameters Measurement

The blood samples collected from male offspring at 24 weeks of age were centrifuged at 3000 × g for 10 min at 4°C, and the serum was stored in aliquots at -80°C. The serum insulin concentrations were measured using an ELISA kit (80-INSMSU-E01, Salem, NH, United States). Insulin sensitivity was assessed using the homeostasis model assessment of insulin resistance (HOMA-IR). The HOMA-IR was calculated as previously described (Zheng et al., 2014). Serum total cholesterol (TC), triacylglycerol (TG), high-density lipoprotein cholesterol (HDL-C), low-density lipoprotein cholesterol (LDL-C) and free fatty acids (FFA) were measured by routine automated laboratory methods.

### Gut Microbiota Analysis

Microbial DNA was extracted from the caecal contents using a QIAamp DNA Stool Mini Kit (Qiagen, Hilden, Germany) according to manufacturer’s protocols. The V3-V4 regions of the 16S rRNA genes were amplified using the primers 341F 5’-CCTACGGGRSGCAGCAG-3′and 806R, 5′-GGACTACVVGGGTATCTAATC-3′. Amplicons were purified using the AxyPrep DNA Gel Extraction Kit (Axygen Biosciences, Union City, CA, United States) and quantified using Qubit^®^2.0 (Invitrogen, United States). The tags were sequenced on the Illumina HiSeq platform (Illumina, Inc., CA, United States).

After merging paired-end reads, reads were performed by quality filtering. High quality reads were clustered into operational taxonomic units (OTUs) with the 97% similarity using UPARSE software (version 7.0.1001) (Edgar, 2013), and representative sequences for each OTU were screened using QIIME software (version 1.7.0, Quantitative Insights into Microbial Ecology) (Caporaso et al., 2010). Then, the GreenGene Database (DeSantis et al., 2006) was used to annotate taxonomic information based on the RDP classifier version 2.2 algorithm (Wang et al., 2007). Alpha and beta diversity analysis were achieved by QIIME software (Version 1.7.0) and R software (Version 2.15.3). For alpha diversity, Chao1, Simpson and the Shannon index were analyzed. For beta diversity, principal coordinates analysis (PCoA) plots and analysis of similarities (ANOSIM) were performed using unweighted UniFrac. In addition, linear discriminant analysis (LDA) of the effect size (LEfSe) was used to determine differences among the groups.

### Statistical Analysis

The data were expressed as mean ± standard error of the mean (S.E.M). The statistics were analyzed by one-way ANOVA and two-way ANOVA, with Turkey *post hoc* analyses. The differences of the relative abundance of gut microbiota were analyzed by Kruskal–Wallis test, with Benjamini and Hochberg *post hoc* analyses. Correlation analyses between the relative abundance of bacterial taxa at genus levels and metabolic parameters were performed by Spearman correlation coefficient test. A *p* value < 0.05 was considered statistically significant. Prism version 7.0 (GraphPad Software Inc., San Diego, CA, United States) was used for statistical analysis.

## Results

### The Effects of Maternal Genistein Intake on Glucose Tolerance and Insulin Sensitivity

**FIGURE 1:**
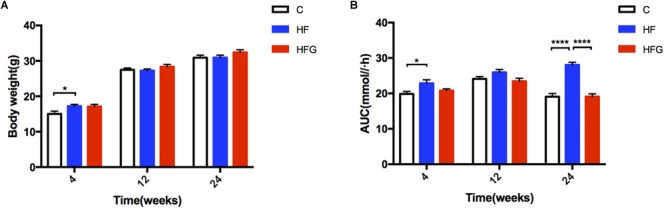
Body weight and AUC of OGTT for male offspring from 4 to 24 weeks. **(A)** Body weight, and **(B)** AUC. C, normal control diet; HF, high-fat diet; HFG, high-fat diet with genistein. Data are expressed as means ± S.E.M. (*n* = 7–8/group). Mean values were significantly different between the groups: ^∗^*p* < 0.05, ^∗∗^*p* < 0.01, ^∗∗∗^*p* < 0.001, ^∗∗∗∗^*p* < 0.0001.

**FIGURE 2:**
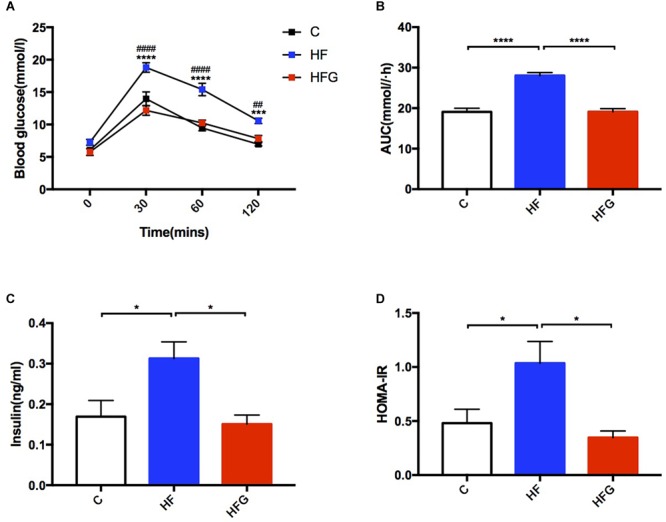
Glucose metabolism of the male offspring at 24 weeks of age. **(A)** OGTT; **(B)** AUC; **(C)** serum insulin levels; **(D)** HOMA-IR. C, normal control diet; HF, high-fat diet; HFG, high-fat diet with genistein. OGTT, oral glucose tolerance test; AUC, area under the curve; HOMA-IR, the homeostasis model assessment of insulin resistance. Data are expressed as means ± S.E.M. (*n* = 7–8/group). Mean values were significantly different between C group and the HF group during OGTT: ^∗^; Mean values were significantly different between HFG group and the HF group during OGTT: ^#^. Mean values were significantly different between the groups: ^∗^*p* < 0.05, ^∗∗^*p* < 0.01, ^∗∗∗^*p* < 0.001, ^∗∗∗∗^*p* < 0.0001.

Consistent with higher body weight in male offspring of the dams fed a HF diet compared with that of C fed dams (*p* < 0.05) at 4 weeks of age (Figure 1A), they had significant glucose intolerance at this time (Figure 1B). After fed a normal chow diet, there was no significant difference of body weight among the three groups throughout the study. However, at the end of the study, there was significantly impaired glucose tolerance in the adult offspring of the HF fed dams (*p* < 0.0001), which was almost normalized in mice of dietary genistein fed dams (*p* < 0.0001) (Figure 1B). As shown in Figure 2, the blood glucose levels were higher at 30 min (*p* < 0.0001), 60 min (*p* < 0.0001) and 120 min (*p* < 0.001) (Figure 2A) and the AUC was significantly larger (*p* < 0.0001) (Figure 2B) for offspring of HF group than that of C group at 24 weeks of age. Male offspring in HFG group showed a dramatic improvement in glucose metabolism with lower blood glucose levels at 30 min (*p* < 0.0001), 60 min (*p* < 0.0001) and 120 min (*p* < 0.001) and smaller AUC (*p* < 0.0001) than that in HF group. Moreover, to determine whether maternal genistein influenced insulin sensitivity in adult offspring, we examined the fasting serum insulin levels. It indicated that serum insulin concentrations (*p* < 0.05) and HOMA-IR (*p* < 0.05) of offspring in the HF group were significantly higher than that from C group. Maternal dietary genistein prevented the deleterious effects on insulin sensitivity induced by HF, with significantly lower insulin levels (*p* < 0.05) and HOMA-IR(*p* < 0.05) (Figure 2C,D).

### Maternal Dietary Genistein Counteracted Lipid Metabolic Disorders Induced by Early Life High-Fat Diet in Adult Mice

**FIGURE 3:**
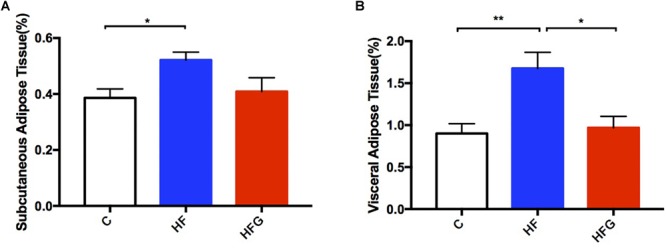
Fat mass of the male offspring at 24 weeks of age. **(A)** Subcutaneous adipose tissue; and **(B)** visceral adipose tissue. C, normal control diet; HF, high-fat diet; HFG, high-fat diet with genistein. Data are expressed as means ± S.E.M. (*n* = 7–8/group). Mean values were significantly different between the groups: ^∗^*p* < 0.05, ^∗∗^*p* < 0.01.

In addition to glucose metabolism, we assessed the effects of maternal dietary genistein on serum lipid profiles and fat mass in adult offspring. The serum lipids of adult offspring among the three groups were summarized in Table 1. Maternal genistein feeding did not alter the levels of TC, LDL-C and FFA in offspring at 24 weeks of age. The levels of serum TG (*p* < 0.01) were significantly higher in offspring of HF fed dams than that from C group, whereas maternal genistein feeding resulted in a dramatic improvement in the serum TG (*p* < 0.01) in male offspring. Furthermore, the levels of HDL-C were also higher in mice of the HFG group compared with that of HF group. Although maternal dietary genistein did not influence the body weight of the adult offspring, we further detected the effects of maternal genistein intake on fat mass in offspring by weighing the adipose tissue. As shown in Figure 3, the **SAT** and **VAT** were both significantly increased in offspring of HF fed dams than that of C fed dams (*p* < 0.05, *p* < 0.01). Maternal dietary genistein dramatically decreased the visceral fat mass induced by maternal HF in adult offspring (*p* < 0.01).

**Table 1:** The lipid metabolic parameters among the three groups.

Lipid profiles	C (*n* = 8)	HF (*n* = 8)	HFG (*n* = 7)
TC (mmol/l)	2.51 ± 0.05	2.83 ± 0.15	2.64 ± 0.04
TG (mmol/l)	0.37 ± 0.02	0.61 ± 0.05^∗∗^	0.41 ± 0.05^##^
HDL-C (mmol/l)	1.56 ± 0.03	1.54 ± 0.04	1.66 ± 0.02^#^
LDL-C (mmol/l)	0.13 ± 0.01	0.16 ± 0.02	0.12 ± 0.01
FFA (nmol/l)	1526.00 ± 113.80	1790.00 ± 99.98	1537.00 ± 59.25

*Data are expressed as means ± S.E.M (*n* = 7–8/group). Mean values were significantly different between the HF group and C group: ^∗^*p* < 0.05, ^∗∗^*p* < 0.01. Mean values were significantly different between the HFG group and HF group: ^#^*p* < 0.05, ^##^*p* < 0.01. C, normal control diet; HF, high-fat diet; HFG, high-fat diet with genistein. TC, total cholesterol; TG, triacylglycerol; HDL-C, high-density lipoprotein cholesterol; LDL-C, low-density lipoprotein cholesterol; FFA, free fatty acids.*

### The Changes of Gut Microbiota in Adult Offspring

**FIGURE 4:**
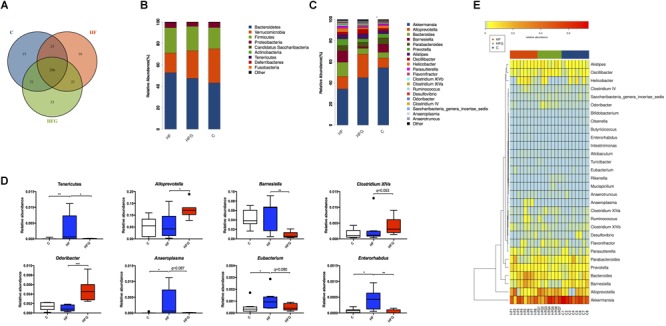
The changes of gut microbiota in adult offspring. **(A)** Venn diagram of the OUTs; **(B)** relative abundance of the top ten phyla; **(C)** relative abundance of the top twenty genera; **(D)** Significantly different germs at the phylum and genus levels; and **(E)** Heat map analysis of the different genera among the three group. C, normal control diet; HF, high-fat diet; HFG, high-fat diet with genistein. Data are expressed as means ± S.E.M. (*n* = 7–8/group). Mean values were significantly different between the groups: ^∗^*q* < 0.05, ^∗∗^*q* < 0.01.

To explore the mechanism that maternal dietary genistein prevented metabolic disorders caused by HF and the changes of gut microbiota, we performed 16S rDNA sequencing for caecal contents in adult offspring among the three groups. The amplicon sequencing data have been submitted to the sequence read archive (SRA) database (accession number PRJNA529750). Firstly, we assessed the OTUs among the three groups to identify the shared and unique species. Venn diagram showed that there were 296 shared OTUs, 15 unique OTUs in offspring of C fed dams, 16 OTUs in mice of HF fed dams, as well as 15 OTUs in offspring of HFG fed dams (Figure 4A). Then we evaluated the composition of the gut microbiota in offspring. At the phylum level, the Bacteroidetes, Firmicutes, Verrucomicrobia and Probacteraia were the most dominant, whereas the relative abundance of the four phyla were not significantly different among the three groups (Figure 4B). The phylum Tenericutes was significantly increased in mice of the HF fed dams compared with that in the C group (*q* < 0.01) (Figure 4D). And maternal genistein feeding could dramatically decreased the abundance of this phyla (*q* < 0.01). Figure 4C showed the relative abundance of top 20 species at the genus level among the three groups. The *Anaeroplasma* (*q* < 0.05), *Eubacterium* (*q* < 0.05) and *Enterorhabdus* (*q* < 0.05) were all significantly increased in male offspring of HF fed dams compared with that of C fed dams. The abundance of *Barnesiella* (*q* < 0.01), *Anaeroplasma*(*q* = 0.087), *Eubacterium* (*q* = 0.080) and *Enterorhabdus* (*q* < 0.01) were distinctly decreased, whereas the *Alloprevotella* (*q* < 0.05), *Clostridium XIVa* (*q* = 0.053) and *Odoribacter* (*q* < 0.001) were increased in offspring of HFG fed dams compared with that in the HF group. Significantly different species at the genus level among the three groups were summarized in a heatmap (Figure 4E).

**FIGURE 5:**
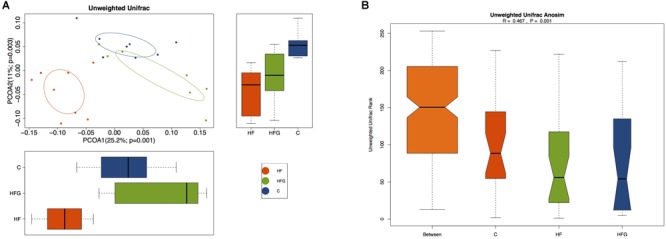
Beta-diversity analysis of the gut microbiota in the adult male offspring. **(A)** PCoA plots of gut communities; and **(B)** Unweighted Unifrac ANOSIM analysis between the three groups (*n* = 7–8/group). C, normal control diet; HF, high-fat diet; HFG, high-fat diet with genistein.

**FIGURE 6:**
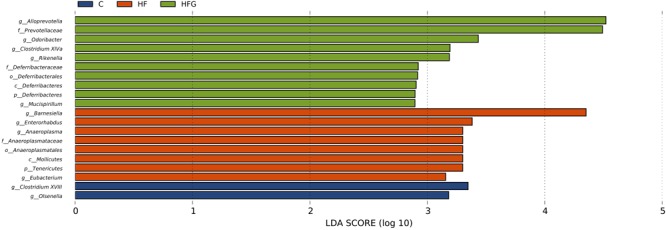
The LEfSe analysis of the different gut microbiota from the phylum level down to the genus level (*n* = 7–8/group). C, normal control diet; HF, high-fat diet; HFG, high-fat diet with genistein.

Then we analyzed the gut microbial structure in mice among the three groups. Alpha diversity analysis revealed that there was a parallel community richness (Chao 1) and diversity (Simpson and Shannon index) among the groups (Supplementary Table S2 and Supplementary Figure S1). Principal coordinate analysis (PCoA) on unweighted UniFrac distances was done to characterize the differences among groups. As shown in Figure 5A, the intestinal microbiota was dramatically separated in adult offspring among the three groups. ANOSIM analysis demonstrated that the differences among the groups were significant (*R* = 0.467, *p* = 0.001) (Figure 5B). Figure 6 lists the significantly different microbiota among the groups at the phylum, class, order, family and genus level. The genus *Clostridium XVIII* and genus *Olsenella* were significantly enriched in the C group. The abundance of genus *Barnesiella*, genus *Enterorhabdus*, genus *Anaeroplasma* from the phylum Tenericutes and genus *Eubacterium* were distinctly increased in mice of the HF fed dams. Maternal dietary genistein significantly enriched the genus *Alloprevotella* from family *Prevotellaceae*, genus *Odoribacter*, genus *Clostridium XlVa*, genus *Rikenella* and genus *Mucispirillum* from phylum Deferribacteres in adult offspring. Thus, maternal genistein feeding changed the composition and abundance of gut microbial communities in male offspring.

### Correlation Analyses Between Gut Microbiota and Metabolic Parameters

**FIGURE 7:**
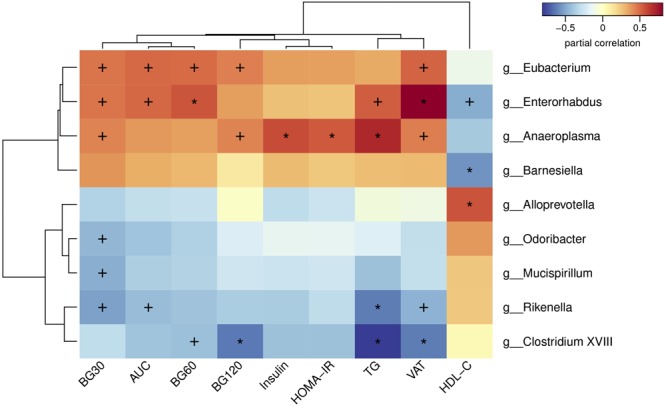
Heatmap of spearman correlation analysis between the altered genera and glucose and lipid metabolic parameters. BG30, blood glucose level at 30 min of OGTT; BG60, blood glucose level at 60 min of OGTT; BG120, blood glucose level at 120 min of OGTT; AUC, area under the curve of OGTT; HOMA-IR, the homeostasis model assessment of insulin resistance; TG, triglyceride; VAT, visceral adipose tissue mass; HDL-C, high-density lipoprotein cholesterol. Values were significantly correlated between the genera and glucose and lipid metabolic parameters: ^∗^*p* < 0.05; ^+^*p* < 0.01.

In order to analysis the relationship between gut microbiota and glucose and lipid metabolism in adult offspring, we performed a correlation analysis between the changed metabolic parameters and significantly different microbiota at the genus level (Figure 7). The blood glucose level during the OGTT and AUC were positively correlated with the relative abundance of *Eubacterium, Enterorhabdus*v and *Anseroplasma*, but were negatively correlated with the abundance of *Odoribacter, Mucispirillum, Rikenella* and *Clostridium XVIII* (*p* < 0.05 or *p* < 0.01). In light of the insulin sensitivity, fasting insulin levels (*p* < 0.01) and HOMA-IR (*p* < 0.01) were both positively correlated with the genus *Anaeroplasma*. In addition to glucose metabolism, we found that the serum TG was positively correlated with the abundance of *Enterorhabdus* (*p* < 0.05) and *Anseroplasma* (*p* < 0.01), but were negatively correlated with the *Rikenella* (*p* < 0.01) and *Clostridium XVIII* (*p* < 0.01). The **VAT** mass also had significantly positive correlation with the relative abundance of *Eubacterium* (*p* < 0.05), *Enterorhabdus*v (*p* < 0.01) and *Anseroplasma* (*p* < 0.05), whereas had negative correlation with *Rikenella* (*p* < 0.05) and *Clostridium XVIII* (*p* < 0.01). By contrast, the serum HDL-C level was negatively correlated with *Enterorhabdus*v (*p* < 0.05) and *Barnesiella* (*p* < 0.01), but was positively correlated with*Alloprevotella* (*p* < 0.01).

## Discussion

During the last few decades, a multitude of studies have demonstrated that adverse early-life exposures were associated with the prevalence of obesity and diabetes. Consistent with published works (Zheng et al., 2014; Cuthbert et al., 2017), our present study also showed that maternal high-fat diet for 3 weeks prior to pregnancy and throughout pregnancy and lactation resulted in significant glucose intolerance, insulin resistance and disorders of serum lipid profiles in adult male offspring. Thus, early life might be a critical window for fighting against the rapid increase of metabolic disorders. It has been commonly accepted that genistein has beneficial effects on glucose homeostasis and lipid metabolism (Gilbert and Liu, 2013). However, research on the influence of early-life genistein intake on metabolism in later life is limited. In the current study, we found that maternal dietary genistein during pre-pregnancy, pregnancy and lactation could significantly counteract the deleterious effects induced by high-fat diet on glucose tolerance, insulin sensitivity and lipid metabolism in adult offspring. Our previous studies and other experimental animal models only detected the protective effects of maternal genistein feeding on metabolism in early life of offspring (Zhang et al., 2015; Han et al., 2017; Zhou et al., 2018). To the best of our knowledge, this is the first study showing the benefits of genistein supplementation in early life on improving metabolic health in adult offspring.

Recent evidence has shown that gut microbiota might play important roles in linking adverse early-life environments and metabolic disorders in later life (Codagnone et al., 2019). Given the crucial role of gut microbiota in metabolic health and the relationship between genistein intake and gut microbial community, we hypothesized that there were alterations in intestinal microbiota in adult mice experienced by early-life genistein exposures. The results revealed that maternal high-fat diet resulted in significant dysbiosis of gut microbial composition and structure, whereas maternal genistein feeding counteracted these changes.

Our present studies showed that early-life over-nutrition (high-fat diet) significantly increased the relative abundance of the phylum Tenericutes as well as the genus *Barnesiella*, genus *Enterorhabdus*, genus *Anaeroplasma* from the phylum Tenericutes in male offspring, which were positively correlated with the levels of blood glucose during OGTT, AUC of OGTT, insulin and TG, but negatively correlated with HDL-C. Similarly, Yan et al. (2016) demonstrated that the Tenericutes were higher in type 2 diabetic rats than lean controls at the phylum level. Furthermore, the phylum Tenericutes have been shown to be increased in HFD fed mice and are related with obesity-associated metabolic parameters (Lecomte et al., 2015). *n* - 3 PUFA deficiency has been shown to be associated with development and progression of a multitude of chronic metabolic disorders. The relationship between *n* - 3 deficient diets and elevated Tenericutes phylum and *Anaeroplasma* has been preliminary confirmed (Robertson et al., 2017). The *Enterorhabdus* and *Barnesiella* and were also be implicated in the development of T2DM, obesity and non-alcoholic fatty liver disease in humans or mice (Le Roy et al., 2013; Yang et al., 2015). More specifically, low-birth-weight mice experienced by nutrition restriction during late pregnancy with accelerated postnatal growth also leaded to significant increase of *Enterorhabdus* and *Barnesiella* in infants (Wang et al., 2016). Thus, the dramatically elevated Tenericutes, *Barnesiella, Enterorhabdus* and *Anaeroplasma* might played vital roles in deciphering the deleterious metabolic effects of maternal high-fat diet on adult offspring. Therefore, decreasing the abundances of those species could bring some metabolic benefits. It has been reported that prebiotic, such as inulin and xylooligosaccharide, which had beneficial effects on metabolic health, could decrease the abundance of Tenericutes and *Enterorhabdus* (Yang et al., 2015; Zhang et al., 2018; Li et al., 2019). In this study, we found that maternal genistein intake could significantly reduce the abundance of Tenericutes, *Barnesiella, Enterorhabdus* and *Anaeroplasma* in adult offspring, which might play crucial roles in mediating the transgenerational metabolic benefits of dietary genistein.

Maternal dietary genistein intake significantly enriched the *Alloprevotella, Odoribacter* and *Clostridium XlVa* at the genus level in adult offspring, which were all confirmed to be short chain fatty acid (SCFA) producers (Pryde et al., 2002; Gomez-Arango et al., 2016; Shang et al., 2017; Shi et al., 2018). A large number of studies has illustrated that the abundance of these bacteria were lower in obese or type 2 diabetic rats or humans (Schwiertz et al., 2010; Gomez-Arango et al., 2016; Jung et al., 2016). By contrast, improvement of glucose and lipid metabolism by polyunsaturated fatty acids (Li et al., 2018), berberine fumarate (Cui et al., 2018), dietary fucoidan (Shang et al., 2017), oral hydroxysafflor yellow A (Liu et al., 2018), a leucine-deprived diet (Wei S. et al., 2018), a galacto-oligosaccharide-rich diet (Cheng et al., 2018) or a traditional Chinese medicine prescription-Xiexin Tang (Wei X. et al., 2018) were all positively correlated with the abundance of these bacterial species. SCFAs (mainly acetate and butyrate), which were produced by fermentation of indigestible dietary components by specific gut microbiota, appeared to promote epithelial restitution and have anti-inflammation activity (Sturm and Dignass, 2008). The main roles of SCFAs were to regulate glucose homeostasis and insulin sensitivity in the liver, skeletal muscle and adipose tissue, possibly though inhibiting intracellular lipolysis to decrease the lipid overflow and ectopic accumulation of fat and regulating chronic inflammation by activating anti-inflammatory Treg cells or directly decreasing the production of proinflammatory cytokines and chemokines (Tremaroli and Backhed, 2012; Canfora et al., 2015). Similarly, in our present study, the enrichment of these three SCFA-producing genera in adult mice of HFG fed dams were negatively correlated with the unfavorable glucose and lipid metabolic parameters. It might be that their metabolites SCFAs possessed the metabolic protective effect. However, the specific mechanisms of the altered bacteria regulating metabolism still need further exploration.

In addition, the genus *Rikenella* from the family *Rikenellaceae* was also significantly enriched in adult offspring of genistein fed dams. Although the concrete function of *Rikenella* remained largely unclear, the opposite relationship between the abundance of *Rikenella* or *Rikenellaceae* with body weight or fat mass in mice and humans has been demonstrated in several studies (Oki et al., 2016; Li et al., 2017). Our previous research also showed that maternal dietary genistein intake could significantly increase the abundance of *Rikenella* in early life of offspring (Zhou et al., 2018). Correlation analysis indicated that improved glucose and lipid metabolic parameters were significantly associated with elevated *Rikenella* in mice of genistein fed dams. Thus, our studies demonstrated that the genus *Rikenella* might be a crucial factor in mediating the beneficial effects of maternal genistein intake in offspring metabolism throughout the life.

In conclusion, maternal dietary genistein intake significantly mitigated the adverse effects of high-fat diet on glucose and lipid metabolism in adult male offspring. In the mean time, the changes of gut microbiota might play a crucial role in the metabolic benefits. To the best of our knowledge, this is the first study to show the important role of gut microbiota in linking maternal genistein supplementation and metabolic protects in adulthood. However, there are still several limitations in this study. First, we only investigated the effects of maternal genistein feeding on metabolism in adult male offspring. The female offspring is still worth to be researched in future. In addition, only relationship between the gut microbiota and metabolism was analyzed in this study, while the causality is in request for further exploration. Finally, the specific mechanism by which maternal genistein intake regulates metabolism and gut microbiota in offspring is still unclear and needs further exploration. Overall, our study provides new evidence and methods for offsetting the poor effects of adverse early-life exposures on metabolic health in later life.

## Data Availability

The datasets for this manuscript are not publicly available because the datasets supporting the conclusions of this manuscript are available from the corresponding author on reasonable request. Requests to access the datasets should be directed to XX, xiaoxh2014@vip.163.com.

## Ethics Statement

All the procedures were approved by the Animal Care and Use Committee of the Peking Union Medical College Hospital (Beijing, China, SYXC-2014-0029). All the animal operations were conducted in compliance with the National Institutes of Health Guide for the Care and Use of Laboratory Animals.

## Author Contributions

XX and JZ designed the experiments. LZ and MD performed the experiments. LZ analyzed the data and drafted the manuscript. XX and QZ reviewed the manuscript. All authors approved the submitted version of the manuscript.

## Conflict of Interest Statement

The authors declare that the research was conducted in the absence of any commercial or financial relationships that could be construed as a potential conflict of interest.
