# Supplementary material for: Maternal Genistein Intake Mitigates the Deleterious Effects of High-Fat Diet on Glucose and Lipid Metabolism and Modulates Gut Microbiota in Adult Life of Male Mice
Source: Front Physiol. 2019 Jul 30;10:985. doi: 10.3389/fphys.2019.00985 (PMC6682633; doi:10.3389/fphys.2019.00985)

Supplementary Material

Table S1. The nutritional compositions of three types of diet. C, normal control diet; HF, high-fat diet; HFG, high-fat diet with genistein.

Ingredients	C(g)	HF(g)	HFG(g)
Casein	200	258	258
L-Cystine	3	4	4
Corn Starch	397	0	0
Maltodextrin	132	162	162
Sucrose	100	89	89
Cellulose	50	65	65
Soybean Oil	0	0	0
Corn Oil	70	32	32
t-Butylhydroquinone	0.014	0	0
Mineral Mix S10026	0	13	13
MinarelMix	35	0	0
Vitamin Mix	0	13	13
Vitamin Mix	10	0	0
Choline Bitartrate	2.5	2.6	2.6
Lard	0	316.6	316.6
DiCalcium	0	16.8	16.8
Calcium Carbonate	0	7	7
Potassium Citrate, 1	0	21	21
FD&C Blue Dye #1	0	0.06	0.06
Genistein	0	0	0.6
Total	1000	1000	1000

Table S2. Comparison of estimator indices of alpha diversity. Data are expressed as means ± S.E.M (n=7-8/group). C, normal control diet; HF, high-fat diet; HFG, high-fat diet with genistein.

Estimators	C	HF	HFG
Simpson	0.876±0.022	0.928±0.009	0.897±0.020
Chao1	272.2±7.7	278.6±9.7	284.6±5.9

Shannon	4.82±0.16	5.27±0.12	5.09±0.18

Figure S1. Rarefaction Plot of Simpson, Chao1 and Shannon index.

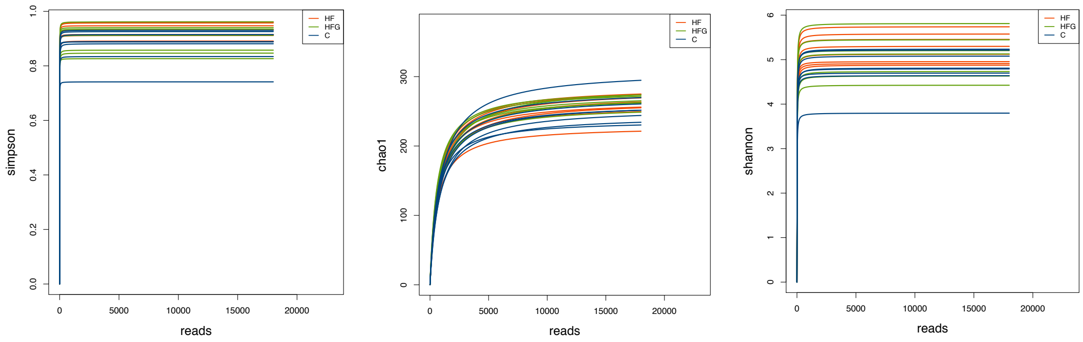

Supplement: Supplementary file 1 [file Data_Sheet_1.PDF]
